# Hypothalamic-Pituitary-Adrenal (HPA) Axis: Unveiling the Potential Mechanisms Involved in Stress-Induced Alzheimer’s Disease and Depression

**DOI:** 10.7759/cureus.67595

**Published:** 2024-08-23

**Authors:** Sharan P, Chitra Vellapandian

**Affiliations:** 1 Pharmacology, SRM College of Pharmacy, SRM Institute of Science and Technology (SRMIST), Chengalpattu, IND; 2 Pharmacy/Pharmacology, SRM College of Pharmacy, SRM Institute of Science and Technology (SRMIST), Chengalpattu, IND

**Keywords:** cortisol, stress, hpa axis, dementia, alzheimer’s disease

## Abstract

The hypothalamic-pituitary-adrenal (HPA) axis plays a pivotal role in the body's response to stress, orchestrating the release of glucocorticoids. In chronic scenarios, these glucocorticoids contribute to various neurological disorders, including Alzheimer's disease (AD) and depression. This abstract explores the potential mechanisms through which HPA axis dysregulation links stress-induced pathways to the pathogenesis of AD and subsequent depression. Chronic stress triggers prolonged HPA axis activation, resulting in elevated cortisol levels, which can lead to hippocampal atrophy, synaptic dysfunction, and neuroinflammation, recognized as key pathological features of AD. These alterations impair cognitive function and may exacerbate amyloid-beta plaque formation and tau hyperphosphorylation, hallmarks of AD. Concurrently, persistent cortisol elevation affects the prefrontal cortex and limbic structures, contributing to depressive symptoms. The interplay between chronic stress, HPA axis dysregulation, and neuroinflammation is crucial in understanding the comorbidity of AD and depression. Unveiling these mechanisms provides insights into potential therapeutic targets aimed at modulating the HPA axis and reducing stress-induced neurodegeneration, offering a dual benefit in managing both AD and depression. Further research is essential to elucidate the precise molecular pathways and develop effective interventions to mitigate the impact of chronic stress on brain health.

## Introduction and background

Alzheimer's disease (AD) is recognized as the leading cause of dementia, significantly affecting global health and economies. The disease's pathophysiology is marked by the buildup of amyloid-beta (Aβ) peptides and tau proteins, which contribute to cognitive decline and physical impairment. Key risk factors for AD include genetic predisposition, advancing age, and a family history of the condition, along with links to other chronic diseases such as diabetes and cardiovascular issues. Depression is a frequent coexisting condition in AD, seen in 20%-30% of patients, but the exact nature of the relationship between depression and AD is still debated. Research has produced mixed results on whether depression serves as a risk factor [1].

Stress is defined as a condition of mental strain or worry brought on by a challenging circumstance. When stress is detected, the hypothalamus releases corticotropin-releasing hormone (CRH), prompting the pituitary gland to secrete adrenocorticotropic hormone (ACTH), which stimulates the adrenal glands to release cortisol. Cortisol prepares the body to handle stress by increasing blood sugar, suppressing the immune system, and enhancing metabolism. As cortisol levels rise, they signal the hypothalamus and pituitary to reduce CRH and ACTH, gradually lowering cortisol levels. Once the stressor is resolved, the body returns to its baseline state, restoring normal functions. Chronic stress can disrupt this balance, but with effective management, the hypothalamic-pituitary-adrenal (HPA) axis can recover, ensuring the body returns to its pre-stress state. It is a basic human reaction that spurs us to confront obstacles and perils in our lives. Everyone goes through periods of stress. The primary mechanisms in humans and many other mammals that react to stress are the autonomic nervous system and the HPA axis. Humans normally release the hormones cortisol and adrenaline in response to stress. Concurrently, the body's return to equilibrium is facilitated by the parasympathetic nervous system.

The HPA axis, another key physiological stress-response system, manages the release of cortisol, affecting various bodily functions, including metabolic, psychological, and immunological processes. Numerous brain areas, including the limbic system, prefrontal cortex, amygdala, hypothalamus, and stria terminalis, regulate the sympatho-adrenomedullary system (SAM) and HPA axes [2]. These mechanisms enable stress to impact memory functions, reward systems, immune responses, metabolism, and disease susceptibility. Over a lifetime, more than 50% of individuals will experience an emotional disorder, often as a result of chronic, untreated stress. According to the 2015 National Health and Morbidity Survey (NHMS) survey, one in every 20 Indians, or 5.3% of the population, was found to have suffered from depressive disorders [3]. It is also one of the top 10 leading causes of death. The majority of dementia cases are caused by AD, and one-third of individuals with mild cognitive impairment (MCI) progress to AD within five years. The development of dementia involves multiple pathways, including modifiable factors that can be categorized into (1) midlife brain health factors such as hypertension, obesity, smoking, and physical activity; (2) cognitive ability and reserve, such as education; (3) testing performance versus central damage, such as hearing loss; and (4) prodromal or reverse causation factors such as depression, social isolation, and physical inactivity, which can vary across different life stages. Consequently, there has been a shift toward primary prevention, with a current focus on treatment as a means of prevention. Cognitive disorders, including AD, infections, problems with the pulmonary and circulatory systems that reduce the amount of oxygen reaching the brain, vitamin B12 deficiency, tumors, and other factors, can all contribute to the progressive deterioration of cognitive abilities.

Depression is characterized by a low mood and a reluctance to engage in activities. It impacts over 280 million individuals worldwide, encompassing all age groups, which is approximately 3.5% of the global population. Individuals with depression frequently experience a lack of motivation or interest in activities that would typically bring them pleasure or joy, leading to diminished enjoyment from these experiences [4]. There is an increasing demand for strategies aimed at early intervention and, ideally, the prevention of psychiatric disorders. However, the evidence supporting the beneficial effects of exercise interventions in preventing depression varies significantly across various studies [5]. Depression can result from chemical changes in the brain and often has a genetic component running in families. It can be triggered by life events or certain illnesses, but it can also develop without an obvious external cause. The hypothalamus, pituitary gland, and adrenal glands interact directly and indirectly through the HPA axis. It is necessary for controlling the body's stress response as well as several physiological systems, including energy management, mood, emotions, immunological response, digestion, and reproductive activities [6]. Due to the lack of review of the relationship between AD and depression, we have identified a novel pathway in the HPA axis. These approaches can be beneficial for researchers and neurobiologists to develop novel therapeutic targets for dementia-induced AD. This review explores the molecular mechanism and therapeutic target of the HPA axis.

## Review

HPA axis dysfunction

The buildup of Aβ plaques and the hyperphosphorylation of tau proteins in the brain are the hallmarks of AD. Neurodegeneration is the final consequence of these degenerative alterations, which cause memory and other cognitive abilities to gradually deteriorate. Dysregulation of the HPA axis persists over time in AD. This axis is regulated by corticotropin-releasing factor (CRF), which is secreted by the hypothalamus. ACTH is released from the anterior pituitary gland in response to CRF, and this, in turn, triggers the adrenal cortex's production of glucocorticoids, which are human cortisol and rat corticosterone. One of the main features of AD is the dysregulation of neuroendocrine functions, particularly in the HPA axis. This imbalance adds to cognitive deterioration and quickens the course of the disease. The central HPA axis is active in the early stages of AD pathology, prior to the manifestation of behavioral symptoms and cognitive impairment. Young mice with 3xTg-AD show an activated HPA axis during early-stage neuropathology, even though their glucocorticoid levels are normal. This activation occurs when mice show mild behavioral changes, suggesting that neuroendocrine regulation is ongoing before severe AD-like pathology and behavioral deficits appear. When exposed to periodic cognitive stimulation, animals show increased levels of corticosterone and Aβ pathology. This suggests that HPA axis dysfunction may cause stimulatory environments to become stressful, leading to a cycle that contributes to Aβ release and plaque formation [7]. The early buildup of abnormal forms of Aβ in humans probably causes this activation, which adds to the general dysregulation of the HPA axis. When AD mice are used as models, this dysfunction shows up as high baseline levels of cortisol in the blood and corticosterone in human patients. According to this theory, long-term exposure to elevated cortisol levels may cause neuronal damage, memory and cognitive decline, and an aggravation of the behavioral signs linked to AD.

The brain's neuronal dysfunction and synaptic loss may be facilitated by the dysregulation of the HPA axis and subsequent overproduction of cortisol as AD progresses. Over time, it is thought that this chain of events exacerbates the behavioral changes and cognitive deterioration seen in AD patients. The dorsal raphe nucleus (DRN) is a brain region that houses serotonergic neurons, which play a crucial role in regulating mood, behavior, and various physiological processes. These neurons send projections to several key brain regions involved in emotional regulation, stress responses, and mood states. Some of the notable brain regions that receive projections from the DRN include the following: (1) The amygdala is known for its role in emotional processing. It receives serotonergic input from the DRN. This connectivity is important for modulating fear responses and emotional memories; (2) the hippocampus is essential for learning, memory, and spatial navigation. It also receives serotonergic innervation from the DRN. This interaction is involved in regulating mood and emotional responses, as well as in the formation of new memories; and (3) the cerebral cortex, particularly regions such as the prefrontal cortex, receives serotonergic input from the DRN. This connectivity is critical for cognitive functions, decision-making, and the regulation of emotions and behaviors [8].

Peroxides and free radicals are produced when a cell's natural redox state is disturbed, which can have harmful consequences. These chemicals are highly reactive and can harm the DNA, lipids, proteins, and other components of cells. The main effects of oxidative stress from cellular metabolism are breakage in DNA strands and damage to DNA bases. Reactive oxygen species (ROS) such as H_2_O_2_, OH, and O_2_ are mostly responsible for indirect base damage [9]. Increased oxidizing species formation or a marked decline in the efficiency of antioxidant defenses such as glutathione are indicative of oxidative stress. ROS are produced more often under stress, which causes oxidative damage to neurons. This oxidative damage further accelerates the underlying degenerative processes of depression and AD.

Neurotransmitter systems

Neurotransmitter system abnormalities, particularly in the norepinephrine and serotonin systems, have been related to depression and chronic stress. These abnormalities cause deficits in cognition and emotional disorders. A class of antidepressants called selective serotonin reuptake inhibitors (SSRIs) is widely utilized; these drugs selectively prevent serotonin from being reabsorbed [10]. L-tryptophan, an amino acid that is converted to serotonin, is carried over the blood-brain barrier via an active transport mechanism. There are two primary types of serotonergic pathways in the brain: descending projections from the caudal raphe nuclei into the spinal cord and ascending projections from the medial and dorsal raphe nuclei. The locus coeruleus and the lateral tegmental area are the main brain regions where noradrenergic neurons are mainly found. The higher norepinephrine content in the synaptic cleft caused by serotonin and norepinephrine reuptake inhibitor (SNRI) administration increases locus coeruleus neuronal activity; α2 adrenergic receptors are activated as a result.

The hypothalamus releases CRH in response to various stimuli, including stress, exercise, disease, blood cortisol levels, and circadian rhythm. After waking up, cortisol levels rise swiftly in healthy people and peak in 30 to 45 minutes. Cortisol progressively falls over the day and rises again in the late afternoon. Cortisol levels begin to decline in the late evening and peak in the middle of the night. This pattern corresponds to the rest-activity cycle of the organism. Distinctly flattened diurnal cortisol levels have been linked to chronic fatigue syndrome, burnout, and insomnia [11]. Many of the body's homeostatic functions, including the immunological, metabolic, cardiovascular, reproductive, and central neurological systems, are controlled by the HPA axis. It integrates psychological and physiological elements to support an organism's survival ability, effectively use resources, and adapt to its surroundings. The pathophysiology of many mood disorders and functional illnesses, including major depressive disorder, burnout, fibromyalgia, irritable bowel syndrome, anxiety, bipolar disorder, insomnia, post-traumatic stress disorder (PTSD), borderline personality disorder, attention deficit hyperactivity disorder (ADHD), and alcoholism, are significantly influenced by the HPA axis. Antidepressants, which are frequently recommended for a number of these ailments, aid in controlling HPA axis activity. When it comes to psychiatric stress-related disorders, women are diagnosed with anxiety and depression more often than men, which is a significant gender disparity [12]. Numerous stress types and their effects on the HPA axis under varied situations have been investigated in experimental research. In rat studies, stressors can be classified as "physical stress" or "social stress," and they both trigger the HPA axis via distinct mechanisms.

**Figure 1:**
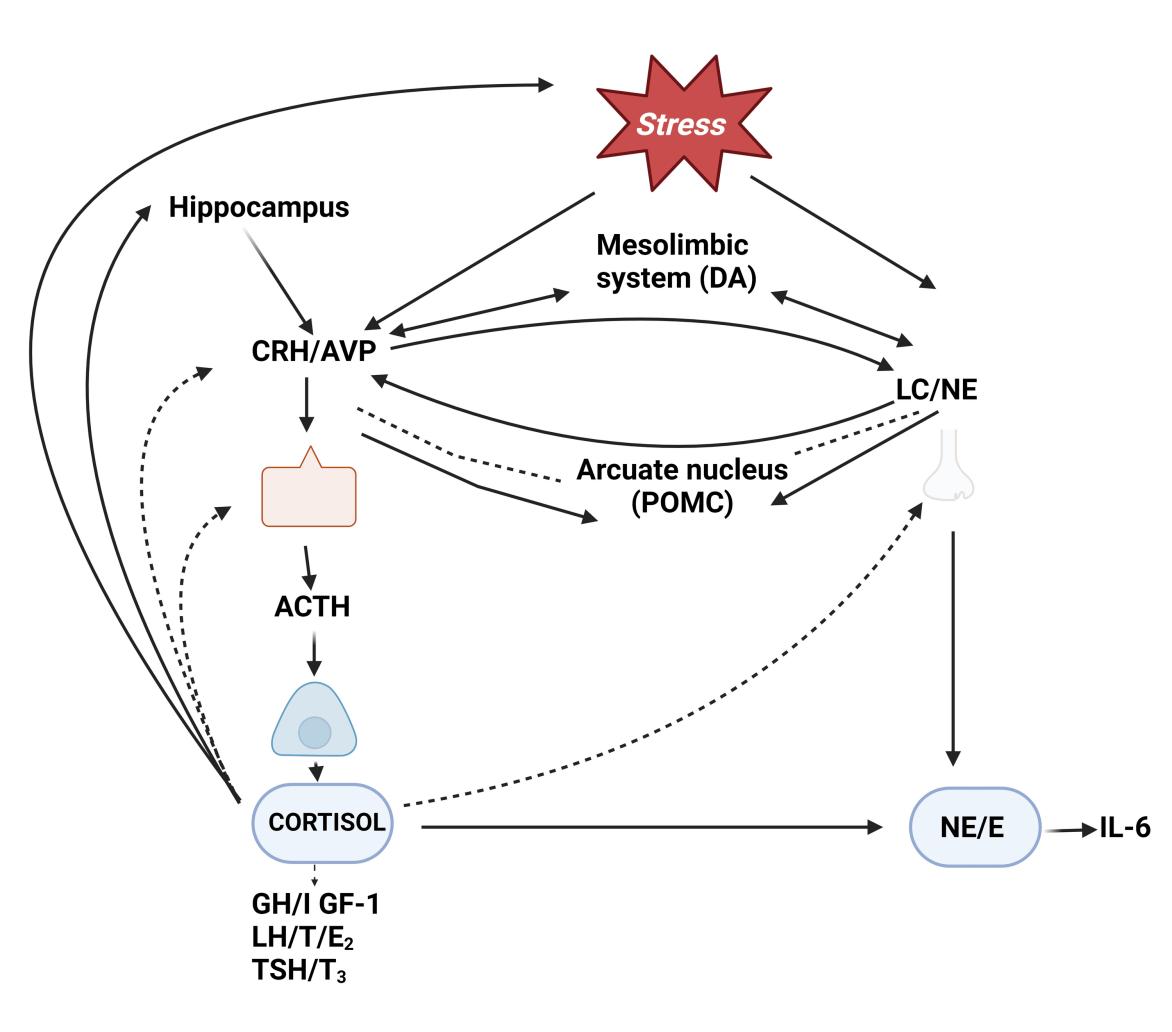
Hypothalamic-pituitary-adrenal (HPA) axis dysfunction The figure is an original illustration of the author, created by Biorender.com. CRH: corticotropin-releasing hormone; AVP: arginine-vasopressin; LC: locus coeruleus; NE: noradrenergic; ACTH: adrenocorticotropic hormone; GH: growth hormone; IL: interleukin; TSH: thyroid-stimulating hormone; LH: luteinizing hormone.

The HPA axis is regulated mainly by monoamine neurotransmitters, specifically dopamine, serotonin, and norepinephrine (noradrenaline). Research indicates that a rise in oxytocin from fulfilling social relationships might inhibit the HPA axis, reducing stress and fostering advantageous health outcomes, including the healing of wounds. There is proof that perinatal stress can affect the regulation of the HPA axis. Prenatal stress exposure has been shown in animal trials to induce a hyper-reactive HPA stress response. In maturity, rats exposed to prenatal stress display aberrant circadian rhythms of corticosterone and higher baseline levels of the hormone. Furthermore, following both brief and persistent stressors, it takes longer for the stress hormone levels of fetal stressed animals to return to baseline. They also have diminished numbers of glucocorticoid receptors in the hippocampus and unusually high blood glucose levels [13]. When the right circumstances are present, humans can partake in behaviors such as eating and sex that advance their intellectual and emotional development and help ensure the survival of their species. However, when they encounter unmanageable risks, the stress reaction is set off, which can result in discomfort and even physical or mental illness. Overexposure to physical or mental stress can lead to a relatively uniform and nonspecific adaptation response in an individual, a phenomenon Selye dubbed "the general adaptation syndrome." It is now known that these adaptive responses are somewhat stressor-specific at first, but that specificity decreases with increasing stressor severity. Stress causes the brain to focus on the perceived threat and sharpen attention. This leads to enhanced respiration and cardiac output, increased catabolism, and rerouting of blood flow circulation to provide enough oxygen and fuel supply to the heart, muscles, and brain during periods of alertness (Figure 1).

Long-term stress system activation is predicted to decrease bone and muscle mass, promote visceral fat accumulation, and prevent the production of new bones. Curiously, this pattern of central obesity and decreased lean body mass is also seen in patients with Cushing's syndrome and some people with metabolic syndrome (i.e., visceral adiposity, insulin resistance, dyslipidemia, and hypertension) in addition to melancholic depression or chronic anxiety disorder [14].

Mechanisms

Numerous biological media, including saliva, cerebrospinal fluid (CSF), perspiration, hair, and urine, can be used to assess cortisol levels. The circadian cycle of this hormone is demonstrated by its early morning peak and midnight minimum. A lesser amount of cortisol in the bloodstream binds to albumin, but the majority of it binds with great affinity to transcortin or corticosteroid-binding globulin (CBG). Approximately 3%-10% of the cortisol in the blood is still free and unbound. One reliable measure of blood levels of free cortisol is salivary cortisol. Its many benefits make it popular in psych neuroendocrinological evaluations: it measures free cortisol noninvasively, does not hurt, and makes it simple for people to easily collect samples at their convenience. Balance control is an advanced motor skill that requires the integration of sensory information, as well as the preparation and performance of adaptive movements.

Cortisol

**Figure 2:**
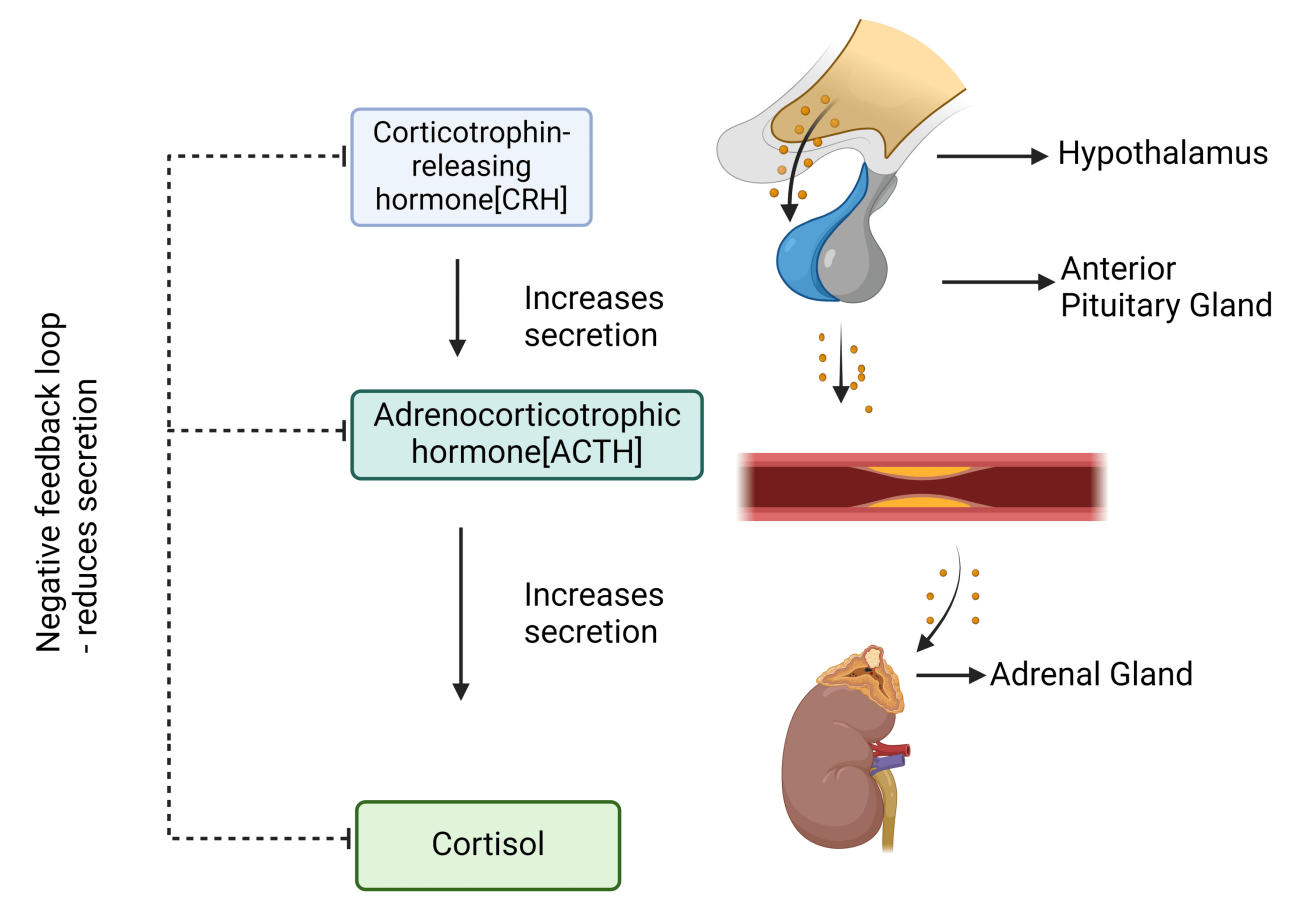
Cortisol Cortisol plays an indirect role in the breakdown of glycogen into glucose-1-phosphate and glucose in the liver and muscles, a process known as glycogenolysis. The main hormones influencing this process are adrenaline and glucagon. For adrenaline to effectively promote glycogenolysis, the enzyme glycogen phosphorylase must be activated, and cortisol will help. The figure is an original illustration of the author, created by Biorender.com.

It is produced in a variety of mammals, mostly by the zona fasciculata located in the adrenal cortex [15]. Its release increases in response to stress and low blood sugar, and it is secreted in a diurnal rhythm. Its functions include immune system suppression, aiding in the metabolism of lipids, proteins, and carbs, and increasing blood sugar through gluconeogenesis. Many of these processes occur when cortisol attaches to glucocorticoid or mineralocorticoid receptors inside the cell. After attaching to DNA, these receptor complexes affect the expression of certain genes. By promoting the synthesis of glucose (gluconeogenesis) and glycogen (glycogenesis) in the liver as well as the breakdown of glycogen (glycogenolysis) in skeletal muscle, cortisol is crucial for controlling glucose metabolism. Through several processes, including decreased glucose uptake in fat cells and muscles, inhibition of protein synthesis, promotion of lipolysis (the breakdown of fats into fatty acids), and the release of amino acids from muscles to assist gluconeogenesis, cortisol elevates blood glucose levels. These processes work together to raise blood glucose levels, which are essential for giving the brain and other tissues the energy they need during stressful reactions such as the fight-or-flight response (Figure 2) [16].

Amyloid beta

The majority of individuals think that Aβ oligomers are the most dangerous kind. Several genetic, cell biology, biochemical, and animal investigations employing experimental models bolster the notion that Aβ is central to the pathogenesis of AD. How Aβ builds up in the central nervous system and damages cells remains unknown. Understanding the processes behind the production of Aβ has been the subject of much research, with particular attention paid to the functions of the proteolytic enzyme's gamma- and β-secretases, which change amyloid precursor protein (APP) into Aβ. The most amyloidogenic version of the peptide, Aβ42, is more hydrophobic than the other forms. On the other hand, the fibril core, or central sequence KLVFFAE, has the ability to generate amyloid on its own. A study that linked the development of AD to brain Aβ42 levels and a reduction in CSF fluid pressure suggests that the pathophysiology of the illness may be impacted by the buildup or insufficient clearance of Aβ42 fragments. The relationship between amyloid beta and the HPA axis involves a complex feedback loop. Chronic HPA axis activation raises cortisol levels, which increases Aβ production and plaque formation, contributing to AD. Conversely, Aβ accumulation disrupts HPA axis function, leading to further cortisol dysregulation. This interaction exacerbates neuroinflammation and cognitive decline, particularly affecting the hippocampus [17].

Neuroinflammation

Inflammation in the nervous system is known as neuroinflammation, and it can be brought on by several conditions, including infections and traumatic brain injuries. It is commonly accepted that neuroinflammation affects the central nervous system on a chronic rather than an acute basis. Acute inflammation is characterized by the presence of inflammatory chemicals, endothelial cell activation, platelet deposition, and tissue edema and usually happens right after central nervous system damage [18]. There is no peripheral immunological response at first. On the other hand, persistent inflammation eventually causes tissue deterioration and the blood-brain barrier to fail. Microglia create ROS and send out signals that enlist the help of peripheral immune cells to take part in the inflammatory response at this time. Microglia, astrocytes, endothelial cells, and other glial cells are among the many cell types in the brain that generate cytokines and chemokines. These compounds control growth and inflammation by acting as neuromodulators. Normal brain function involves the secretion of cytokines by brain cells to trigger a localized inflammatory response, which in turn attracts microglia to remove wounds or infections.

**Figure 3:**
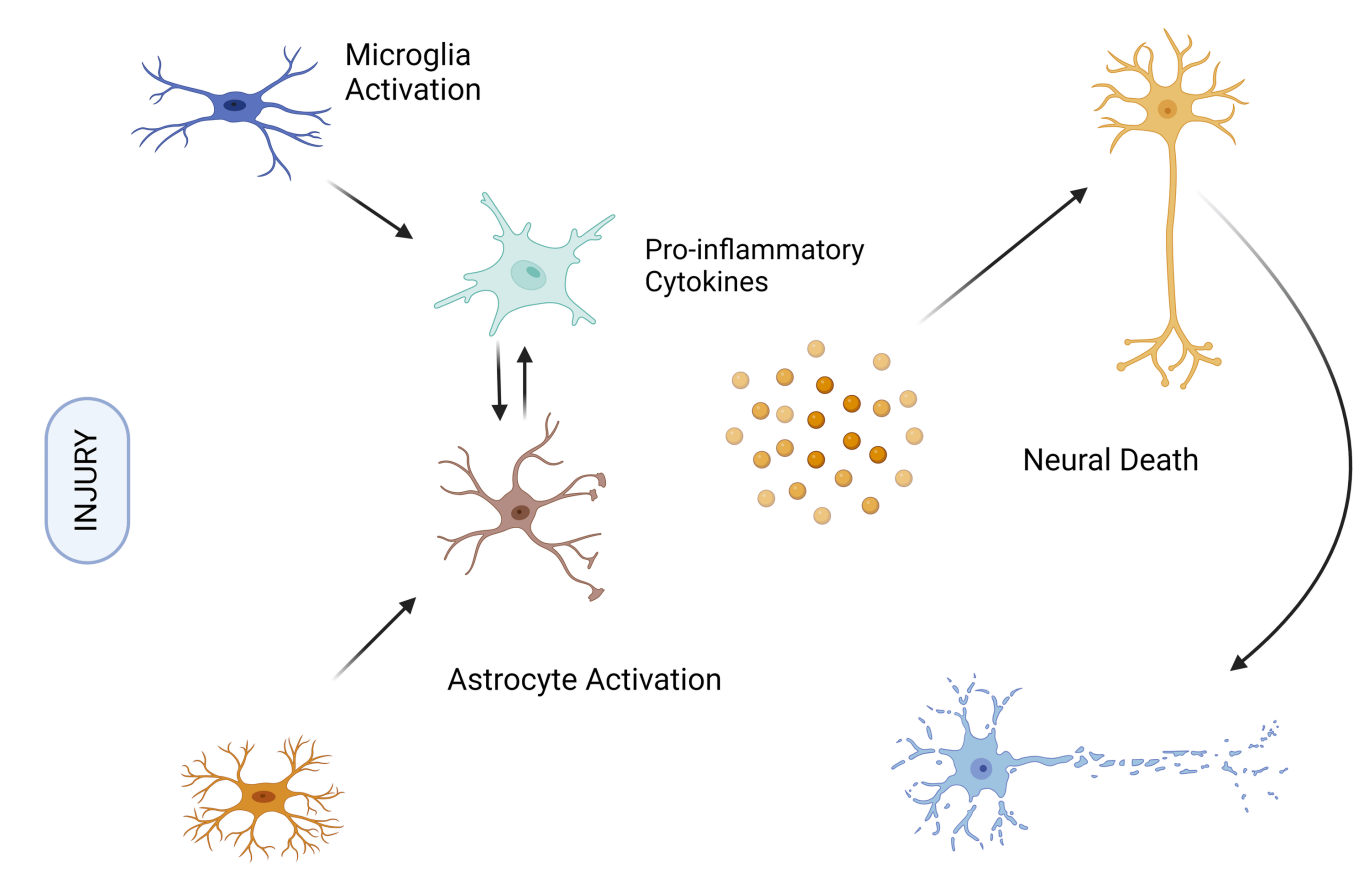
Neuroinflammation The figure is an original illustration of the author, created by Biorender.com.

Nevertheless, cytokines and chemokines may be released for an extended period of time during neuroinflammation, which may jeopardize the blood-brain barrier. These cytokines attract peripheral immune cells to injury sites, where they can move into the brain across the weakened blood-brain barrier. Interleukin-6 (IL-6), which is created during astrogliosis, and interleukin-1 beta (IL-1β) and tumor necrosis factor alpha (TNF-α), both of which can cause neuronal cytotoxicity, are often produced cytokines in response to brain damage. Pro-inflammatory cytokines are necessary for the healing of damaged tissue, but they can also cause cell death and further tissue damage. Activated microglia are notably present in the post-mortem brains of AD patients. It is currently thought that these microglia are less efficient at phagocytosing Aβ when stimulated by inflammatory cytokines. Rather than helping to eliminate Aβ plaques from the brain, it is believed that this reduced capacity to remove Aβ contributes to their buildup. Increased levels of the inflammatory cytokine IL-1β are linked to decreased synaptophysin levels and consequent synapse loss in AD. This emphasizes the role that inflammation plays in the development of AD. Non-steroidal anti-inflammatory medicine (NSAID) users have demonstrated a 67% lower incidence of AD compared to placebo recipients in a four-year follow-up trial, which lends further credence to this association (Figure 3) [19].

Neuroplasticity

Impaired neuroplasticity, the brain's ability to change and reorganize itself, is a feature of both illnesses. Neuroplasticity can be reduced by long-term stress and HPA axis dysregulation, which can have detrimental effects on mood regulation and cognitive performance. They called this process "long-term potentiation" since the postsynaptic potential lasted far longer than expected. According to the idea, the postsynaptic neuron responds to input from the presynaptic neuron by growing the number of neurotransmitter receptors, which lowers the threshold for activation from the presynaptic neuron. Exercise, the environment, task repetition, motivation, neuromodulators such as dopamine, and specific treatments or pharmaceuticals can all have a favorable impact on synaptic plasticity. This kind of neuroplasticity frequently looks at how various internal or external stimuli affect the anatomical reorganization of the brain. Structural neuroplasticity includes modifications to the amount of gray matter present in the brain or the strength of synapses. Structural neuroplasticity is currently one of the main areas of academic neuroscience research concentration. The brain's ability to modify and adjust the functional characteristics of neurons is known as functional plasticity. BDNF (brain-derived neurotrophic factor) is crucial for neuronal plasticity, aiding in synaptic strengthening, neuronal survival, and the formation of new synapses, especially in areas such as the hippocampus involved in learning and memory. It also regulates neurotransmitter release, which influences cognitive functions and mood. Adequate BDNF levels support cognitive health, while reduced levels are linked to neurodegenerative and psychiatric disorders. Activities such as exercise can boost BDNF, therefore enhancing brain resilience against stress and cognitive decline. There are four recognized mechanisms to facilitate this kind of plasticity: compensating masquerade, cross-modal reassignment, map expansion, and homologous area adaptation [20].

A cognitive task is moved from a damaged brain region to its comparable area on the opposite side of the brain in a process known as homologous area adaptation. Compared to adults, children are more likely to experience such functional alterations. Map expansion is the result of repeated exposure to stimuli, which causes cortical maps associated with particular cognitive processes to enlarge. Experiments involving individuals learning spatial routes have been used to demonstrate the effect of repeated inputs on brain functional connectivity. Synaptic plasticity refers to alterations in synapses that are reliant on activity. Long-term potentiation (LTP) is the process by which synapses get stronger, increasing the rate at which neurons fire; long-term depression (LTD) is the weakening of synapses that results in a decrease in the neuronal firing rate. LTD and LTP are two instances of synaptic plasticity associated with memory.

In addition to synaptic plasticity, intrinsic plasticity, a different type of activity-dependent plasticity, has also been shown to be important in recent times. Intrinsic plasticity modifies the intrinsic excitability of neurons, in contrast to homeostatic plasticity, which seeks to preserve total neural activity within a network. This suggests that rather than just modifying the overall firing rates of neurons to maintain network equilibrium, it also plays a role in memory encoding by changing the neurons' reactivity to stimuli.

Management

Non-pharmacological Management

Lifestyle medicine, focusing on preventing and treating diseases through healthy behaviors, emphasizes nutrition, physical activity, sleep, stress management, and social connection. A balanced diet rich in whole, plant-based foods and low in processed items, regular exercise such as aerobic workouts, strength training, and yoga, quality sleep with a regular schedule, and stress management through mindfulness, meditation, and deep breathing are all essential components. Strong social ties, achieved through community engagement and maintaining relationships, also play a critical role in mental and cognitive health. For AD, alternative approaches include nutraceuticals and supplements such as omega-3 fatty acids, curcumin, and ginkgo biloba, which support brain health. Herbal medicines such as *Bacopa monnieri* and *Ashwagandha* offer cognitive-enhancing and stress-reducing benefits, respectively [21]. Mind-body interventions such as yoga, Tai Chi, and meditation improve cognitive function and reduce stress. Cognitive training, through puzzles, reading, and memory exercises, helps maintain cognitive abilities, while environmental and sensory therapies, such as music and art therapy, provide emotional and cognitive benefits. Depression can be managed through nutritional psychiatry, emphasizing an anti-inflammatory diet and maintaining a healthy gut microbiome with probiotics and prebiotics. Herbal medicines such as St. John's wort and *Rhodiola rosea* are effective for mild to moderate depression and fatigue [22]. Mind-body therapies such as mindfulness-based stress reduction (MBSR) and cognitive behavioral therapy (CBT) focus on reducing stress and changing negative thought patterns. Regular physical activities are also beneficial for alleviating depression symptoms. An integrative approach combining conventional treatments with lifestyle changes and alternative therapies offers a holistic strategy for managing AD and depression. Consulting healthcare professionals to create a personalized plan is crucial for effective treatment.

**Figure 4:**
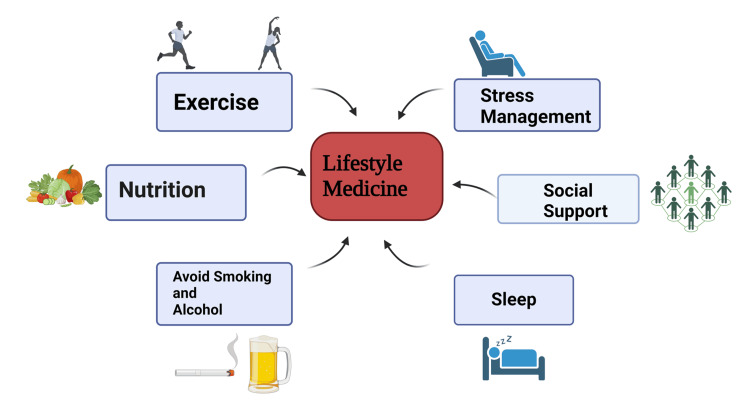
Lifestyle medicine The figure is an original illustration of the author, created by Biorender.com.

**Figure 5:**
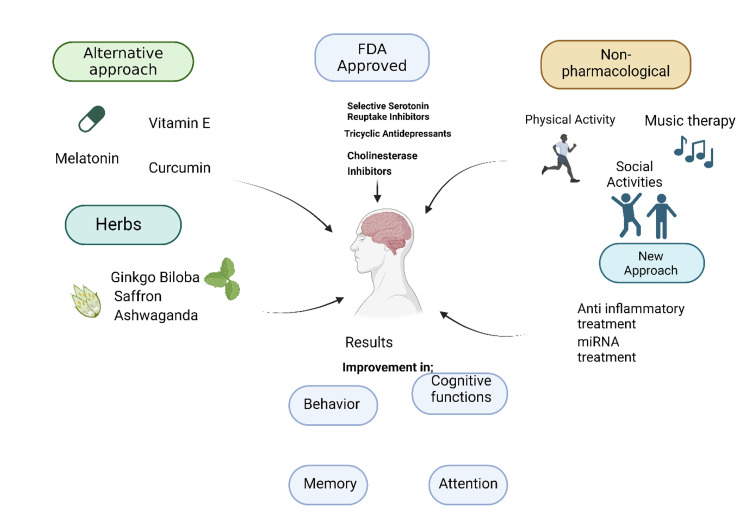
A new and alternative approach to the treatment of AD and depression The figure is an original illustration of the author, created by Biorender.com.

The therapeutic approach is similar to lifestyle medicine and constitutes a new approach to treating AD and depression (Figures 4, 5).

Pharmacological Management

SSRIs such as fluoxetine, sertraline, and paroxetine inhibit the HPA axis. For instance, fluoxetine SSRIs work by serotonin reuptake transporter (SERT), leading to increased serotonin levels in the synaptic cleft. Fluoxetine enhances cognitive function by improving mood and reducing depression-related impairments. It increases BDNF, supporting neuroplasticity and neurogenesis, particularly in the hippocampus. Additionally, its modulation of serotonin helps with memory and cognitive flexibility. This enhancement of serotonergic neurotransmission helps alleviate symptoms of depression and anxiety. Fluoxetine, an SSRI, affects the HPA axis through multiple mechanisms. Its primary action involves blocking serotonin reuptake in the brain, indirectly influencing the HPA axis. Serotonin is crucial in regulating CRF release from the hypothalamus, a key hormone in the HPA axis. By modulating serotonin levels, fluoxetine can alter CRF secretion, thereby impacting the stress response system. Additionally, fluoxetine's promotion of neuroplasticity and neurogenesis in certain brain regions may contribute to its ability to modulate the HPA axis, potentially mitigating the effects of chronic stress. Studies have also suggested that fluoxetine treatment can lead to changes in glucocorticoid levels, such as cortisol, which are regulated by the HPA axis. These complex interactions highlight fluoxetine's broader therapeutic effects beyond its antidepressant properties, potentially influencing conditions where HPA axis dysregulation is implicated, such as anxiety disorders and other stress-related conditions.

SNRIs such as venlafaxine, desvenlafaxine, and duloxetine inhibit the HPA axis. For instance, venlafaxine is primarily prescribed as an antidepressant within the class of SNRIs. It is effective in treating conditions such as major depressive disorder, generalized anxiety disorder, panic disorder, and social anxiety disorder. However, its direct impact on cortisol levels, specifically within the context of the HPA axis, is not well-documented or a primary focus of its clinical application. Venlafaxine primarily inhibits the reuptake of serotonin and norepinephrine in the brain, affecting mood regulation. While these neurotransmitters can indirectly influence the HPA axis, the medication's intended therapeutic effects are centered around managing mood disorders rather than directly altering cortisol levels. If there are concerns regarding how venlafaxine may interact with cortisol in a specific medical context, it is crucial to consult a healthcare provider for personalized advice and management.

Tricyclic antidepressants (TCAs) such as amitriptyline, nortriptyline, and imipramine are commonly prescribed to treat major depressive disorder, anxiety disorders, and neuropathic pain. Unlike newer antidepressants such as SSRIs and SNRIs, TCAs primarily work by inhibiting the reuptake of serotonin and norepinephrine in the brain. Antidepressants such as TCAs, SSRIs, and SNRIs affect BDNF levels, which are important for brain health and mood regulation. TCAs indirectly increase BDNF through elevated neurotransmitter levels, although their effect is less clear. SSRIs consistently boost BDNF by increasing serotonin levels, while SNRIs raise BDNF by elevating both serotonin and norepinephrine. The rise in BDNF linked to these medications is thought to contribute to their antidepressant effects. However, its direct impact on cortisol levels within the HPA axis is not well-documented or a primary focus in clinical settings. The therapeutic effects of amitriptyline are centered around its modulation of neurotransmitter systems to alleviate symptoms of depression, anxiety, and pain. While changes in mood and anxiety levels influenced by amitriptyline treatment may indirectly affect stress responses and cortisol levels, any such effects are secondary to its primary pharmacological actions. Therefore, if there are specific concerns about how amitriptyline might interact with cortisol or the HPA axis in an individual case, consulting a healthcare provider is essential for personalized evaluation and management.

Monoamine oxidase inhibitors (MAOIs) such as phenelzine, tranylcypromine, and isocarboxazid are primarily prescribed as antidepressants but can also affect the HPA axis, which regulates cortisol levels. MAOIs such as phenelzine alter neurotransmitter levels, potentially impacting the stress response and cortisol production. This can lead to changes in cortisol levels, although the specific effects can vary among individuals. Healthcare providers monitor cortisol levels when using phenelzine to manage its impact on the HPA axis and assess any related effects on hormone regulation. It is crucial for patients on phenelzine to adhere closely to their prescribed regimen and consult with their healthcare team regarding any concerns about hormone levels or medication effects.

Examples of cholinesterase inhibitors are donepezil, rivastigmine, and galantamine. Donepezil, a cholinesterase inhibitor used to treat AD, may impact the HPA axis and cortisol levels by inhibiting acetylcholinesterase. Donepezil increases acetylcholine levels, which can stimulate the release of CRH from the hypothalamus. CRH promotes ACTH release from the pituitary gland, leading to cortisol production in the adrenal glands. This cholinergic activity might alter cortisol secretion, with effects varying by dosage, treatment duration, and individual differences. Additionally, donepezil's cognitive benefits could reduce stress, potentially normalizing HPA axis activity and cortisol levels. While primarily used for AD, donepezil's influence on the cholinergic system and cortisol levels suggests potential therapeutic effects for conditions involving HPA axis dysregulation, warranting further research [23].

Examples of Aβ-targeting therapies are aducanumab, lecanemab, and donanemab. Aducanumab, a monoclonal antibody approved for AD, targets Aβ plaques in the brain to slow disease progression. By reducing amyloid plaques and associated neuroinflammation, aducanumab may help restore normal HPA axis function and cortisol regulation. Chronic neuroinflammation can disrupt the HPA axis, leading to abnormal cortisol levels, so improving neuronal health might normalize cortisol secretion. Additionally, aducanumab's potential to slow AD progression and improve cognitive and behavioral symptoms could reduce stress and anxiety, further normalizing HPA axis activity and cortisol levels [24]. While its primary action is on Aβ reduction, understanding aducanumab's broader impact on the HPA axis and cortisol regulation requires further research to explore its potential therapeutic benefits beyond AD treatment.

Discussion

The HPA axis is crucial for the body's reaction to stress, and dysregulation in this pathway has been implicated in both AD and depression. The cortisol pathway, a key component of the HPA axis, becomes particularly relevant when examining AD-induced depression. In the HPA axis, stress triggers the hypothalamus to produce CRH. CRH stimulates the pituitary gland to secrete ACTH, which in turn prompts the adrenal cortex to produce cortisol. Cortisol, the primary stress hormone, facilitates various physiological responses, including modulation of immune function and glucose metabolism. Cortisol levels are normally regulated by negative feedback mechanisms and follow a daily cycle. In AD, this regulatory mechanism often becomes impaired. Elevated cortisol levels are frequently observed in AD patients, suggesting chronic activation of the HPA axis. Elevated cortisol levels can have detrimental consequences on the nervous system, particularly in the hippocampus, a portion of the brain that is vital for memory and emotional control. Thus, the hippocampus also plays a role in the negative feedback control of the HPA axis, and its impairment in AD exacerbates HPA axis dysregulation.

The link between HPA axis dysregulation and depression in AD is multifaceted. Chronic elevated cortisol can lead to hippocampal atrophy, contributing to both cognitive decline and depressive symptoms. Moreover, cortisol affects neurotransmitter systems, including serotonin, norepinephrine, and dopamine, which are crucial in mood regulation. Imbalances in these neurotransmitters are a hallmark of depression. Additionally, inflammation, which is prevalent in AD, can further disrupt HPA axis function and cortisol levels. Pro-inflammatory cytokines can alter HPA axis activity, leading to sustained high cortisol levels and increased vulnerability to depression.

In summary, in AD, the dysregulation of the HPA axis, characterized by chronic cortisol elevation, contributes to both neurodegeneration and the development of depressive symptoms. Understanding this pathway highlights potential therapeutic targets, such as cortisol modulation and anti-inflammatory strategies, to alleviate depression in AD patients.

## Conclusions

Chronic stress can lead to long-term alterations in brain function and structure, particularly affecting areas such as the hippocampus, which is crucial for memory and learning. Depression, particularly in mid-life, is considered a risk factor for developing AD later in life. Depression can exacerbate cognitive decline and memory problems, potentially accelerating the progression from mild cognitive impairment to AD. Chronic stress frequently results in depression, and both conditions are interconnected through shared pathways, especially those involving the HPA axis, which plays a significant role in brain health. This interplay may heighten the risk of AD by worsening neurodegenerative processes and cognitive decline.

The HPA axis is crucial for managing the stress response, and its dysregulation can have significant effects on both AD and depression. Persistent stress can keep the HPA axis activated, resulting in elevated cortisol levels, a key stress hormone. In AD, prolonged exposure to cortisol is linked to increased production of beta-amyloid plaques, which intensify neuroinflammation and damage neurons, especially in the hippocampus, contributing to cognitive decline. Similarly, individuals with depression often experience HPA axis hyperactivity and high cortisol levels, leading to structural brain changes such as reduced hippocampal volume and impaired neurogenesis. This dysregulation is observed in both conditions and is further complicated by interactions between stress, cortisol, and neurotransmitter systems such as serotonin and dopamine. Understanding these HPA axis mechanisms reveals future insights and treatments for individuals at risk for or affected by AD and depression.
